# Engaging in prosocial behavior explains how high self-control relates to more life satisfaction: Evidence from three Chinese samples

**DOI:** 10.1371/journal.pone.0223169

**Published:** 2019-10-14

**Authors:** Kai Dou, Jian-Bin Li, Yu-Jie Wang, Jing-Jing Li, Zi-Qin Liang, Yan-Gang Nie

**Affiliations:** 1 Department of Psychology and Research Center of Adolescent Psychology and Behavior, School of Education, Guangzhou University, Guangzhou, P. R. China; 2 Department of Early Childhood Education, The Education University of Hong Kong, Hong Kong SAR, China; 3 Mental Health Education and Counseling Center, Guangdong Industry Polytechnic, Guangzhou, P. R. China; 4 Institute of Developmental Psychology, Beijing Normal University, Beijing, P. R. China; Middlesex University, UNITED KINGDOM

## Abstract

High levels of self-control are found to be associated with greater life satisfaction. To further understand this relationship, the current study examined two questions: (1) whether too much self-control reduces, rather than increases, life satisfaction, as argued by some scholars; and (2) whether engaging in prosocial behavior explains the “self-control–life satisfaction” link. To this end, we conducted survey research among adolescents (*N* = 1,009), university students (*N* = 2,620), and adult workers (*N* = 500). All participants answered the same self-control and life satisfaction measures, whereas prosocial behavior was assessed using different scales across samples. Results of two-line regressions failed to reveal significant inverted-U shaped association between self-control and life satisfaction across samples. Moreover, results of mediation analyses showed that across samples, high levels of self-control were related to greater life satisfaction and this association was partly mediated by prosocial behavior. In conclusion, there is no evidence showing that too much self-control impairs life satisfaction. Engaging in prosocial behavior partly explains *how* high self-control relates to greater well-being.

## Introduction

Self-control refers to the ability to change one’s thoughts, emotions, and behaviors to comply with social norms and to support goal-directed behavior [1, 2]. While low self-control has long been viewed as a major cause of various emotional and behavioral problems [2–4], researchers recently have realized that there is insufficient understanding on whether and how self-control affects individual’s well-being. This awareness has led to a growing body of studies investigating the relationship between self-control and well-being indicators such as life satisfaction in the past few years [5–9].

However, there are two gaps in the literature that warrant further study. First, while prior research assumes that the relationship between self-control and life satisfaction is linear, some scholars contend that too much self-control (defined as very high levels of self-control) may result in less rather than more life satisfaction [10]. This suggests an inverted-U relationship between self-control and life satisfaction, such that life satisfaction increases as self-control increases, but life satisfaction reduces when self-control exceeds a certain level. However, only few studies have addressed this question [11] and thus further evidence is needed. Second, a recent research found that direct intervention to enhance self-control is not as effective as usually thought [12]. This implies that aiming to boost life satisfaction by improving individuals’ self-control seems not an optimal approach. Instead, examining the mechanisms underlying the “self-control—life satisfaction” association would provide pivotal insight on how to enhance personal life satisfaction. Although several studies have examined this issue [5–9], each mechanism only accounts for a small proportion of the relationship. Hence, investigating other unexplored mechanism is still needed. Taken together, this research aims to (1) examine whether there is an inverted-U association between self-control and life satisfaction, and (2) examine prosocial behavior as a potential, but untested, mechanism.

### Self-control and life satisfaction

Life satisfaction is an overall judgment dependent on one’s satisfaction with the fields important to him/her [13]. Individuals with high self-control are more likely to achieve life goals and have better physical and psychosocial functioning, which possibly leads to a positive appraisal of one’s life [7]. Besides, individuals high in self-control are prone to use positive coping strategies, focus on promotional regulation, perceive optimal levels of job satisfaction, and satisfy basic psychological needs, which are all beneficial to one’s experience of good and happy life [5–9]. All these findings suggest self-control is linearly related to life satisfaction.

While the linear relationship between self-control and life satisfaction prevails in the literature, it is also argued that there may be an inverted-U relationship between the two constructs. Individuals with an under-control profile (i.e., reflected as low-score in the self-control scale) are prone to act without restraint, which may results in externalizing problems; whereas those with an over-control profile (i.e., reflected as very high-score in the self-control scale) are likely to suppress their emotion, which may results in internalizing problems [14]. In this sense, individuals with high levels of self-control are adept at dealing with difficulties, handling interpersonal and motivational conflicts successfully, more often achieving personal goals, and engaging in less externalizing problems, all of which are important etiological sources of long-term satisfaction with life [15]. However, people with very high levels of self-control are likely to overregulate cognition, emotions, and behaviors impairs positive interpersonal relationship [16], brings about rigidity and limits the enjoyment of momentary positive emotion [10], and likely induces excessive anxiety in the process of leading indivdiuals to focus on fulfilling personal goals too much [17]. All these problems could possibly reduce personal life satisfaction [7, 18, 19]. Taken together, this suggests an inverted-U relationship between self-control and life satisfaction.

A recent research has systematically examined the inverted-U shaped association between self-control and life satisfaction in a number of samples [11]. However, results of this study did not find any significant evidence for such an inverted-U shaped association. Despite such early findings, it is important to provide more evidence before drawing a robust conclusion. Hence, the first goal of this study was to explore whether the relation between self-control and life satisfaction is linear or inverted-U shaped.

### The role of prosocial behavior

Prosocial behavior refers to actions that benefit others, such as cooperation, sharing, helping, and caring [20]. In this study, we contend that individuals with high levels of self-control would engage in more prosocial behavior, which in turn associates with greater life satisfaction.

Prosociality is automatic whereas deliberation leads to payoff-maximizing behavior. This phenomenon has also been observed in the domain of cooperation [21], altruism [22], and honesty [23]. While prosocial behavior is promoted in most cultures, motivational conflicts often arise in the process of being prosocial [24]. Overcoming such conflict requires the exertion of self-control, an ability to regulate one’s own dominant responses to align with social norms and values [1, 2]. In other words, successful engagement in prosocial behavior needs the recruitment of self-control. Studies from different fields reconcile to suggest that self-control plays a central role in prosocial behavior. For example, developmental studies have found that adolescents high in self-control report more prosocial behavior [25–27]. Behavioral research has revealed that experimentally manipulating self-control into a state of depletion affects different dimensions of prosociality including prosocial behaviors [24], cooperation in the prisoner's dilemma [28], altruism in the dictator game [29, 30], and honesty in cheating tasks [31]. Work from neuroscience has suggested that brain regions related to self-control are recruited during prosocial behaviors [32].

Theorists have asserted that people who are socially responsible and prosocial live a eudemonic life [33], implying that satisfaction with life may be a function of engagement in prosocial behavior. Three reasons may lend support to this view. First, prosocial behavior leads to better relationship and social competence [34, 35], which are seen as important foundation for people to judge their lives as good and satisfied [36]. Second, prosocial behavior is considered as a tool to avoid and relieve negative feeling such as guilt [37], thereby further translating into satisfaction with life. Third, engaging in prosocial behavior satisfies people’s basic psychological needs [9] and brings people sense of meaning, which is known as a crucial source of happiness [38]. Prior studies have found that individuals who often engage in different forms of prosocial behavior (e.g., helping, prosocial spending) report greater life satisfaction [36, 39, 40].

Taken together, based on the association between self-control and prosocial behavior and the one between prosocial behavior and life satisfaction reviewed above, it stands reason for us to expect that prosocial behavior is a plausible, yet untested, factor linking self-control and life satisfaction.

### The present research

In this study, we expected: (1) there would be a linear association between self-control and life satisfaction, but we would also explore whether there would be an inverted-U relationship between the two constructs, and (2) prosocial behavior would mediate the linear relationship between self-control and life satisfaction. In order to expand the generalizability of our research findings, we conceptually examined these two hypotheses in three samples with different demographics (i.e., middle school students, university students, and adult employees). In each sample, we used different measures to capture prosocial behavior. For instance, we used the prosocial behavior subscale of the Strength and Difficulties Questionnaire to assess adolescents’ prosocial behavior, the Prosocial Tendency Measure to capture university students’ prosocial behavior, and the Organizational Citizen Behavior to tap employees’ prosocial behavior at the work context. Two reasons motivated us to do so. First, each measure is specifically designed to tap prosocial behavior of different samples. Second, prosocial behavior has a wide range of behavioral indicators. If the proposed model can be replicated with different prosocial measures, we are more confident to draw robust conclusion.

## Method

### Participants and procedure

Data were collected from three samples. Sample 1 consisted of 1,009 students (493 male, 516 female; *M*_age_ = 14.72 years, *SD* = 1.67) recruited from four middle schools in Guangzhou, China, between March and April 2017. Sample 2 consisted of 2,620 university students (612 male, 2008 female; *M*_age_ = 21.48 years, *SD* = 1.27) recruited from a large university in Guangzhou, China, between April and June 2017. Sample 3 included 500 Chinese full-time employees (198 males, 302 females, *M*_age_ = 28.91 years, *SD* = 5.27; *M*_length of service_ = 49.37 months, *SD* = 66.61; *M*_annual income_ = 67471.84 RMB, *SD* = 61184) who worked in different industries from various regions of China. They are recruited through online advertisements in exchange for the chance to win 100 RMB. The data were collected between April and July 2017. Demographics of each sample are presented in Table 1.

**Table 1:** Demographic characteristics of the three samples.

Variables	Groups	Sample 1 Adolescents		Sample 2 University students		Sample 3 Employees	
		*N*	%	*N*	%	*N*	%
Gender	Female	493	48.9	2008	76.6	302	60.4
	Male	516	51.1	612	23.4	198	39.6
Grade	Grade 7	162	16.1				
	Grade 8	184	18.2				
	Grade 9	182	18.0				
	Grade 10	190	18.8				
	Grade 11	193	19.1				
	Grade 12	98	9.7				
	Freshman			314	12.0		
	Sophomore			648	24.7		
	Junior			553	21.1		
	Senior			542	20.7		
	Other (i.e., missing)			563	21.5		
Marital status	Unmarried					262	52.4
	Married					235	47.0
	Other (i.e., widowed)					3	.6
Education	High school degree or below					19	3.8
	Bachelor degree					353	70.6
	Graduate degree or above					128	25.6
Total		1009	100	2620	100	500	100

The study was approved by the IRB of Guangzhou University. Regarding university students and employees, they provided written consent before participating in the study. Regarding middle school students who were under 18 years, we sought written consent from their legal guardians and students provided their verbal consent before participation. Participants of sample 1 and 2 completed the survey in paper-and-pencil format while participants of sample 3 answered the survey online.

### Measures

#### Self-control

The Brief Self-Control Scale (BSCS) was used to measure participants’ self-control ability [2]. All participants answered the Chinese version of the BSCS which had been used in Chinese populations and had showed sound psychometric properties [4, 6]. The scale includes 13 items rated on a five-point scale (from “1 = not like me at all” to “5 = very much like me”), with a higher score indicating greater self-control. Cronbach’s α was .78, .80, and .81 for middle school students, university students, and adult employees, respectively.

#### Prosocial behavior

Middle school students’ prosocial behavior was measured with the prosocial subscale of the Chinese version of the Strength and Difficulties Questionnaire (www.sdqinfo.org) [41]. This subscale has five items rated on a three-point scale (from “0 = not true” to “2 = certainly true”), with a higher score indicating more prosocial behavior. Cronbach’s α was .75 in this study.

University students’ prosocial behavior was assessed with the Chinese version of Carlo and Randall’s (2002) Prosocial Tendencies Measure [36, 42]. The scale has 26 items which can be divided into six dimensions (i.e., public, anonymous, altruistic, compliant, emotional, and dire prosocial behaviors). All items are rated on a five-point scale (from “1 = does not describe me at all” to “5 = describes me greatly”), with a higher total score indicating more frequent engagement in prosocial behavior. Cronbach’s α was .92 in this study.

Adult employee’s prosocial behavior was measured with Aryee, Budhwar, and Chen’s (2002) Organizational Citizenship Behavior Scale (OCBS)[43]. This scale has two dimensions, one measuring pro-colleagues and the other measuring pro-organization behavior. The former dimension reflects prosocial behavior at individual level whereas the latter indicates prosocial behavior at organizational level. These two dimensions are highly correlated, showing that those who are pro-colleagues are also likely to be pro-organization [44, 45]. Hence, a total score is summed to represent employees’ prosocial behavior within the organization. Participants answered the Chinese version of the scale [45]. It has 9 items rated on a seven-point scale (from “1 = strongly disagree” to 7 = “strongly agree”), with a higher score indicating more prosocial behavior toward colleagues and the organization. Cronbach’s α was .89 in this study.

#### Life satisfaction

Diener et al.’s (1985) Satisfaction with Life Scale (SWLS) was used to measure participants’ life satisfaction[13]. All participants answered the Chinese version which had been validated in the Chinese population [46]. The scale consists of 5 items rated on a seven-point scale (from “1 = strongly disagree” to “7 = strongly agree”), with a higher total score indicating greater life satisfaction. Cronbach’s α was .85, .88, and .88 for middle school students, university students, and adult employees, respectively.

### Data analytic plan

Multiple analyses were performed for each sample. First, descriptive statistics and correlation analyses were carried out. Second, in order to examine whether there would be an inverted-U shaped association between self-control and life satisfaction, we carried out two-lines analyses through an online APP (http://webstimate.org/twolines) developed by Simonsohn [47]. This test tests two regression models simultaneously with the values of the independent variable (in this case, self-control) before and after a break point. A statistically significant inverted U-shaped was supported if: (1) the slope of the first line before the break point had a positive sign, while the second line had an opposite sign; (2) both slopes were significant. A detailed, step-by-step instruction of this analysis can be obtained online (http://webstimate.org/twolines). Third, mediation models were tested in Hayes’ PROCESS macro (v2.13, Model 4) in SPSS. Self-control, prosocial behavior, and life satisfaction were entered the model as independent variable, mediator, and dependent variable, respectively. Effects obtained from the total effect model (i.e., without including the mediator in the model) and the indirect effect model (i.e., with mediator in the model) were reported. Bootstrapping (*N* = 10,000) was employed and the 95% confidence interval (CI) was used to justify the significance of the mediation effect. If the 95% CI does not include 0, then a significant mediation effect is tenable [48].

## Results

### Descriptive statistics and correlation

Means, standard deviation and correlations of samples 1 to 3 are presented in Table 2. Regarding sample 1, self-control was positively related with prosocial behavior (*r* = .30, *p* < .001) and life satisfaction (*r* = .25, *p* < .001). Prosocial behavior was also positively related with life satisfaction (*r* = .25, *p* < .001). Regarding sample 2, self-control was positively related with prosocial tendencies (*r* = .17, *p* < .001) and life satisfaction (*r* = .26, *p* < .001). Prosocial tendency was also positively related to life satisfaction (*r* = .26, *p* < .001). Regarding sample 3, self-control was positively related with organizational citizenship behavior (*r* = .36, *p* < .001) and life satisfaction (*r* = .20, *p* < .001). Organizational citizenship behavior was also positively correlated to life satisfaction (*r* = .21, *p* < .001).

**Table 2:** Descriptive statistics and correlations of self-control, prosocial behavior, and life satisfaction across the three samples.

Samples	Variables	M	SD	Skewness	Kurtosis	1	2	3
Adolescents	1. Self-control	3.26	.60	.13	.18	-		
	2. Prosocial behavior	2.47	.41	**−**.63	.30	.30***	-	
	3. Life satisfaction	4.34	1.37	**−**.13	**−**.31	.25***	.25***	-
University students	1. Self-control	3.17	.54	.17	.54	-		
	2. Prosocial tendencies	2.58	.51	.06	.71	.17***	-	
	3. Life satisfaction	4.08	1.14	**−**.07	**−**.19	.26***	.26***	-
Employees	1. Self-control	3.32	.59	**−**.10	.03	-		
	2. Organizational citizenship behavior	5.44	.96	**−**.99	2.21	.36***	-	
	3. Life satisfaction	3.96	1.39	**−**.14	**−**.93	.20***	.21***	-

Note.

*** *p* < .001.

### Examination of inverted-U shaped association between self-control and life satisfaction

**Fig 1:**
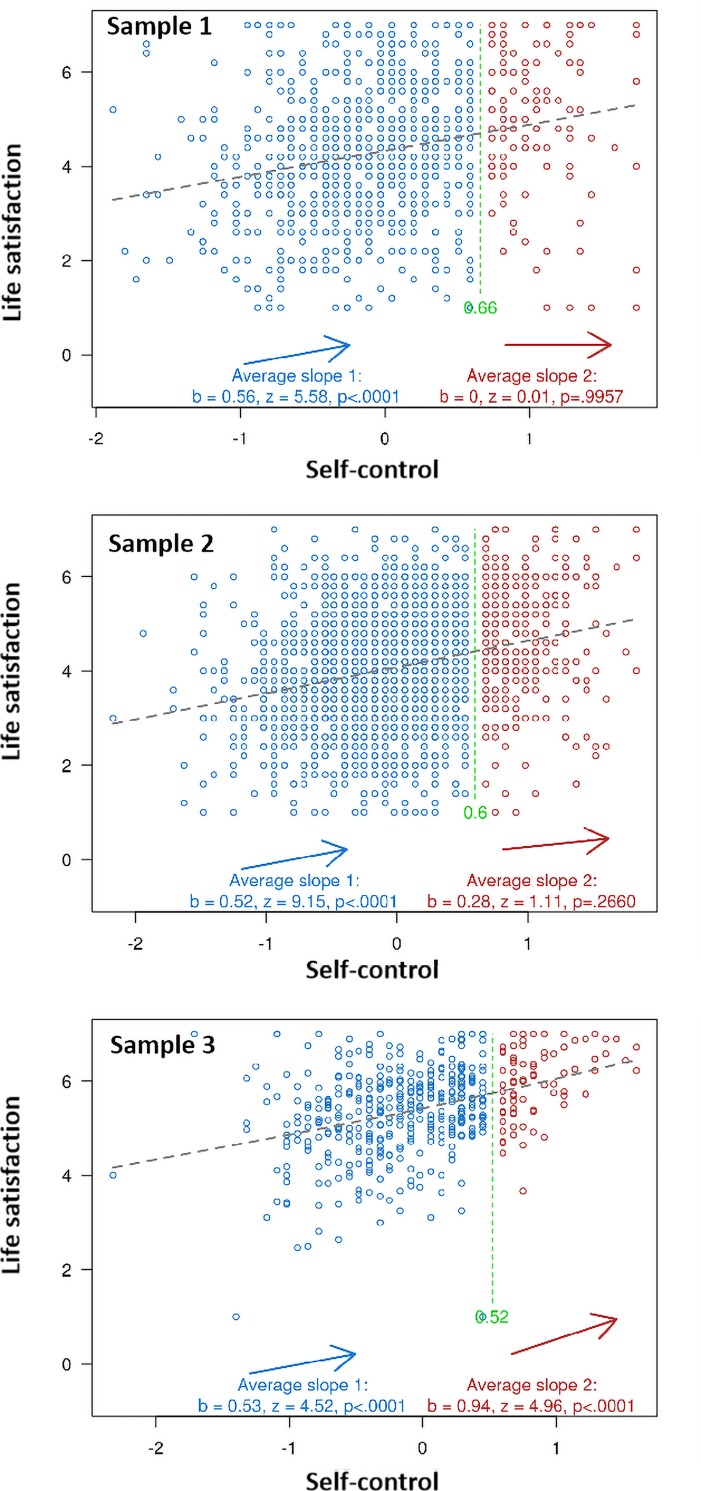
Two-lines test are applied for the relationship between self-control and life satisfaction in three samples.

In order to examine whether there is presence or absence of an inverted U effect of self-control on life satisfaction, we followed Simonsohn’s “two-line” approach [47] and ran the statistics on http://webstimate.org/twolines/. The results are summarized in Fig 1.

Regarding middle school students, the data showed that there was a positive relation between self-control and life satisfaction before the break point (*B* = 0.56, *z* = 5.58, *p* < .001), but the influence of self-control on life satisfaction became insignificant after the break point (*B* = 0.00, *z* = .01, *p* = .996). Regarding university students, a similar result was found, such that the influence of self-control on life satisfaction was significant before the break point (*B* = 0.52, *z* = 9.15, *p* < .001) but it became insignificant after the break point (*B* = 0.28, *z* = 1.11, *p* = .266). Regarding adult employees, the influence of self-control on life satisfaction was significant both before (*B* = 0.53, *z* = 4.52, *p* < .001) and after (*B* = 0.94, *z* = 4.96, *p <* .001) the break point. Taken together, these findings demonstrate that there is no significant evidence showing an inverted-U association between self-control and life satisfaction.

### The mediation effect of prosocial behavior

**Fig 2:**
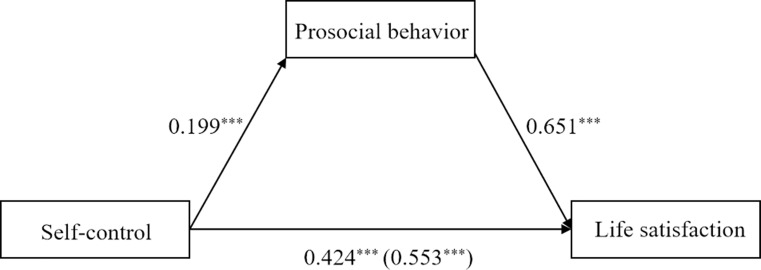
Mediation of prosocial behavior between self-control and life satisfaction in adolescents (sample 1). Note: *** *p*<0.001; value in parenthesis represents total effect.

Regarding middle school students, the total effect model explained 5.98% variance of life satisfaction. The results showed that self-control was positively related to life satisfaction (*B* = 0.55, *SE* = 0.07, *p* < .001). The indirect effect model (Fig 2) explained 9.43% variance of life satisfaction. The association between self-control and life satisfaction reduced but remained significant (*B* = 0.42, *SE* = 0.07, *p* < .001). More importantly, this relation was mediated by prosocial behavior (estimate = 0.13, *SE* = 0.03, 95% bootstrapping CI = [0.08, 0.19]). Controlling for gender and age did not significantly change the results.

**Fig 3:**
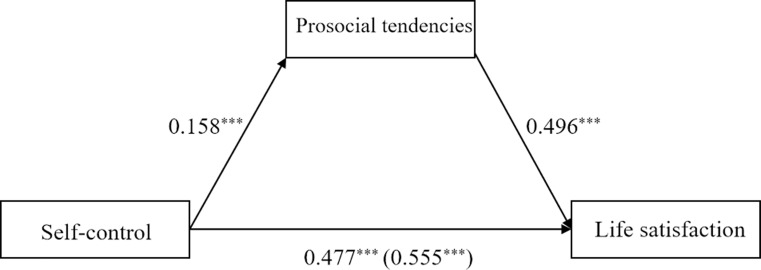
Mediation of prosocial tendencies between self-control and life satisfaction in university students (sample 2). Note: *** *p*<0.001; value in parenthesis represents total effect.

Regarding university students, the total effect model explained 6.87% variance of life satisfaction. The results showed that self-control was positively related to life satisfaction (*B* = 0.56, *SE* = 0.04, *p* < .001). The indirect effect model (Fig 3) explained 11.68% variance of life satisfaction. The association between self-control and life satisfaction reduced but remained significant (*B* = 0.48, *SE* = 0.04, *p* < .001). More importantly, this relation was mediated by prosocial tendencies (estimate = 0.08, *SE* = 0.01, 95% bootstrapping CI = [0.06, 0.10]). Controlling for gender and age did not significantly change the results.

**Fig 4:**
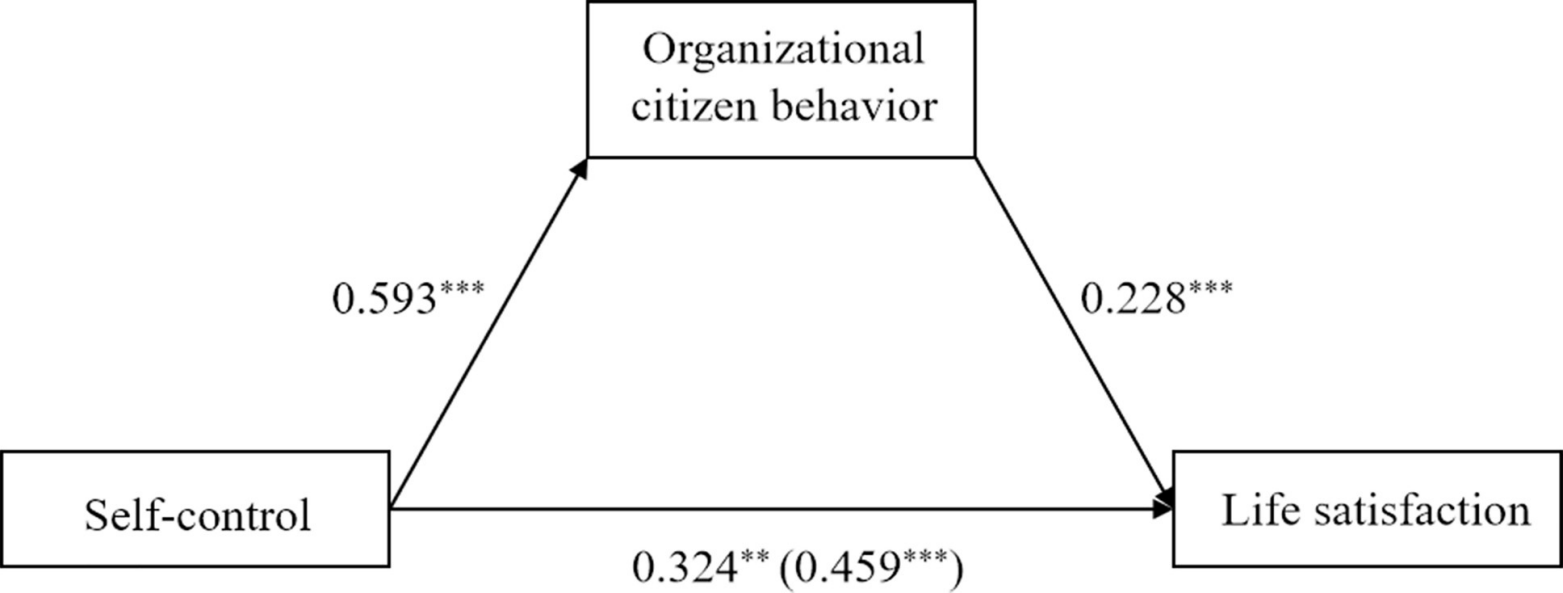
Mediation of organizational citizen behavior between self-control and life satisfaction in employees (sample 3). **p<0.01, ***p<0.001; value in parenthesis represents total effect.

Regarding adult employees, the total effect model explained 3.79% variance of life satisfaction. The results showed that self-control was positively related to life satisfaction (*B* = 0.46, *SE* = 0.10, *p* < .001). The indirect effect model (Fig 4) explained 5.95% variance of life satisfaction. The association between self-control and life satisfaction reduced but remained significant (*B* = 0.32, *SE* = 0.11, *p* = .003). More importantly, this relation was mediated by organizational citizenship behavior (estimate = 0.14, *SE* = 0.05, 95% bootstrapping CI = [0.05, 0.24]). Controlling for gender and age did not significantly change the results.

In each sample, we analyzed the data with and without controlling for gender and age. To save space, we only reported results without controlling for gender and age. In terms of controlling for gender and age, we regressed the mediator and the outcome on both gender and age.

## Discussion

Consistent with prior research [11], our results supported that high levels of self-control were linearly related to greater life satisfaction and that too much self-control did not dampen life satisfaction. Regarding the second question, we disclosed that engaging in prosocial behavior partly explained the relationship between self-control and life satisfaction: people with high levels of self-control have the propensity to engage in more prosocial behavior, which in part translates into higher life satisfaction.

Prior research has examined whether too much self-control is associated with lower rather than higher life satisfaction, and its results suggest a linear rather than inverted-U shaped association [11]. Consistent with this study, our current findings reveals a similar pattern using a different analytic approach. Specifically, our results show that across the three samples, the increase of self-control is related to the increase of life satisfaction before the break point. In two samples (i.e., middle school and university students), continued increase in self-control does not add additional benefit to the gain of life satisfaction. For adult employees, continued increase in self-control still associates with more life satisfaction. Taken together, very high levels of self-control do not impair life satisfaction. If anything, it only stops adding more life satisfaction at most when it reaches a certain level.

Regarding our second question, we found that engaging prosocial behavior partly mediates the relationship between self-control and life satisfaction. Investigation of the influence of self-control on life satisfaction and its related working mechanisms have been only recently picked up [5–9]. The current findings pile on this trendy theme by revealing that prosociality is another important mechanism, too. Although our hypothesized models are confirmed across the three samples, the explained variance differs. This could be probably due to the differences in the characteristics of the samples and in the prosociality measures.

Our findings should be interpreted with caution. First, the data was cross-sectional in nature and were recruited with convenient sampling; thus causality cannot be deduced and generalization of findings is limited. Second, only self-report measures were used, which may cause common method variance. Moreover, the sample sizes as well as the measures used to assess prosociality varied across samples, which renders our findings less comparable across the three different samples. Future study may consider using a prosocial measure that is suitable across samples of different age so that the results can be more comparable. One promising step is scholars could develop a measure to assess prosocial behavior of samples with different demographic characteristics. By this way, future research may conduct multi-group analysis to explore whether the hypothesized model is invariant across samples in a more sophisticated manner. In addition, it is necessary to note that the main variables tested in this study are likely affected by other factors. For instance, the current findings are obtained in the Chinese context. Prior studies have found that self-control, prosocial behavior, and well-being differ among cultures [49–51]. This suggests that the relationships among these constructs could be inequivalent in other cultures. Hence, future studies could consider conducting cross-cultural comparison on the current models. Besides, personal temperament (e.g., negative affect) may be another factor that affects the current variables. For instance, individuals with high levels of negative temperament trait (e.g., anger, neuroticism) are easily in a hotheaded state and susceptible to external stress, which may lead to more self-control failure, less prosocial behavior and life satisfaction [52, 53]. Considering that our findings are drawn from samples recruited in one culture and did not control for temperament factor, we encourage future research to replicate the current findings in different cultural contexts taking possible confounding variables (e.g., temperament) into account.

Nevertheless, this study bears important practical implications. As mentioned above, direct intervention of self-control seems not as effective as usually thought (Friese et al., 2017), and thus programs targeted at increasing life satisfaction by improving self-control may not be optimal. Instead, encouraging individuals to engage in prosocial behavior may be a useful way to enhance one’s life satisfaction, especially for those low in self-control. In fact, prior research already shows that prosocial behavior induced by intervention program increases subsequent life satisfaction [54]. Institutes (e.g., schools and companies) could consider setting up rules and establish climates to encourage daily prosocial behavior, which is a promising avenue to create a happy campus and organization.

## Conclusion

In conclusion, self-control is a great human strength not only protecting against psychopathology but also fostering well-being. Too much self-control does not lower people’s life satisfaction. One mechanism behind the “self-control–life satisfaction” relation is that individuals with high self-control are more willing to benefit others, which in turn associates with more life satisfaction.

## Supporting information

S1 File IRB—protocol number: GZHU2017001 (PDF). (PDF)

S2 File Self-control and life satisfaction–minimized data (zip). (ZIP)
