# Supplementary material for: Engaging in prosocial behavior explains how high self-control relates to more life satisfaction: Evidence from three Chinese samples
Source: PLoS One. 2019 Oct 14;14(10):e0223169. doi: 10.1371/journal.pone.0223169 (PMC6791543; doi:10.1371/journal.pone.0223169)
Supplement: S1 File — (PDF) [file pone.0223169.s001.pdf]

广州大学教育学院
伦理审核证明

Certification of Ethics Review Committee of Education School, Guangzhou University

项目名称: 高自控者为何对自己的生活更满意？亲社会行为的中介机制

Project Title： Engaging in Prosocial Behavior Explains How High Self-Control Relates to Greater Life Satisfaction: Evidence from Three Samples

项目申报人: 窦凯，聂衍刚

Principal/Overall Investigator： Kai Dou, Yangang Nie

批准号： GZHU2017001

protocol number： GZHU2017001

该项目已通过广州大学教育学院伦理委员会的审核，符合伦理原则。

This project is approved by the Ethics Review Committee (IRB) of Education School, Guangzhou University, accords with the principle of ethics.

广州大学教育学院

二零一七年三月十三日

School of Education, Guangzhou University

2017/3/13

付世敏
